# "It's Okay to Say 'I Don't Know'": Medical Students Use Transformative Thinking to Cope with Ambiguity and Uncertainty

**DOI:** 10.15694/mep.2020.000014.1

**Published:** 2020-01-15

**Authors:** Virginia Randall, Charisse Villareal

**Affiliations:** 1 Uniformed Services University of the Health Sciences

**Keywords:** transformative learning, uncertainty, ambiguity, Mezirow, medical student education

## Abstract

This article was migrated. The article was marked as recommended.

Uncertainty refers to the internal tension of not fully knowing or understanding a situation and is a concept that physicians must learn to deal with in order to become an effective provider. Ambiguity refers to the situation that is not fully known. Mezirow proposed that transformative learning occurs in response to a “disorienting dilemma” during which the students’ frames of reference are challenged. We used his model to explain how clerkship students learn to cope with ambiguity and uncertainty. We analyzed third year medical students reflective practice essays for encounters with clinical uncertainty and ambiguity and examined how they reacted to these dilemmas. Our study is unique in its robust data set which included 273 essays.

## Introduction

*“The core predicament of medicine ..is uncertainty..As a doctor, you come to find..that the struggle in caring for people is more often with what you do not know than what you do. Medicine’s ground state is uncertainty. And wisdom - for both patients and doctors - is defined how one copes with it.”* ( Gawande, 2002).

The clerkship year is one of excitement, challenge, disappointment, disillusionment, discovery, reflection and change. It marks the transition from the academic setting of learning from books and lectures to learning from real patients by developing a history and physical examination and moving to a differential diagnosis. It is also often where students are first faced with the reality that medicine is filled with uncertainty and ambiguity.

There is a difference between ambiguity and uncertainty. Ambiguity describes a situation in which the answer is not clearly known; in our clinical settings, laboratory and imaging results may be ambiguous or there may be conflicting results. Uncertainty describes the individuals’ reaction to the ambiguous situation; the individual has difficulty deciding what is the best course of action for the patient. In trying to make more sense and order in the world, uncertainty arises when patient presentation produces a sense of helplessness in a provider. ( O’Riordan *et al.*, 2011). Uncertainty may lead to frustration, loss of self-confidence, and burn-out.

Tolerance of ambiguity and uncertainty affect a provider’s clinical judgment, relationship with a patient, and clinical decisions. ( Luther and Crandall, 2011). Medical students who were less likely to tolerate ambiguity were found to have decreased attitudes towards underserved populations ( Wayne *et al.*, 2011). Ability to tolerate uncertainty may also influence what specialty students choose to pursue ( Merrill *et al.*, 1994). A recent article points to another source of uncertainty experienced by clerks, that of chronic sleep deprivation which makes even fairly straightforward circumstances seem ambiguous and causes the student to question their own judgment ( Taylor, Raynard and Lingard, 2019). Ability to deal with uncertainty, therefore, is an essential skill that providers must have and the foundation for this skill is developed during medical school.

At the Uniformed Services University, Bethesda, MD (USU), students face ambiguity in their own lives such as future deployments, strategic implications of congressionally mandated budget cuts, and more immediately, the sites at which they will do their clerkships. They are often gone from USU for 12 weeks at a time, and lose the support of family and the comforting familiarity of USU. Issues of work-life balance become more difficult. They may be uncertain about their choice of residency specialty and location.

Jack Mezirow began to publish his work on *Transformative Learning* in 1977. Since that time there has been a large body of work from several disciplines exploring the theory as applied to adult education ( Mezirow, 1997). Mezirow describes 10 phases of learning that become clarified in the transformative experience:

1. A disorienting dilemma
2. Self-examination
3. A critical assessment of assumptions
4. Recognition of a connection between one’s discontent and the process of transformation
5. Exploration of options for new roles, relationships, and action
6. Planning a course of action
7. Acquiring knowledge and skills for implementing one’s plan
8. Provisional trying of new roles
9. Building competence and self-confidence in new roles and relationships
10 A reintegration into one’s life on the basis of conditions dictated by one’s new perspective

We became interested in how students learn about and begin to cope with clinical ambiguity during the workshop *Collaborating around Threshold Concepts in Health* (CATCH), in Dundee, Scotland, in June 2019. The topic was to nominate important threshold concepts necessary for medical students to acquire prior to graduation and form the foundation to function as a physician. The phrase “Medicine isn’t black or white but mostly gray” recurred many times in the data from two sources: in the USU pediatric clerks essays ( Randall, McNally, and Wait, 2019) and from audio files from the medical students at Plymouth (Collett, Neve, and Stephen, 2017). The workshop determined that there are two overarching important concepts: the uncertainty of medicine and the importance of patient-centered/community-centered care. We determined to investigate the circumstances under which students first confront uncertainty, the strategies they develop to cope, and the outcome of those efforts. Threshold concepts were first described by Drs. Land and Meyer ( Meyer, 2003) who expanded on the idea of Mezirow’s transformative learning to emphasize the role of ontological change, irreversibility of the phenomenon, the integrative function of a threshold concepts, and the troublesomeness of the approach to and through the threshold of understanding. Rather than focus on the data supporting the concept of the threshold concept in our data ( Randall, McNally, and Wait, 2019), we decided to describe more fully the circumstances under which the students learn how to acknowledge and cope with uncertainty.

We found using the theory of Transformative Learning to be a useful lens through which to examine the students’ encounters with ambiguity and uncertainty.

## Methods

With IRB approval, we analyzed 273 essays from pediatric clerks from academic years 2016 and 2018. The essays were required in the clerkship, were ungraded, and were de-identified for this research. Prompt questions included: What was the most difficult concept you have encountered? What changes have you seen in yourself or in your approach to medicine and learning?

We used a structured content approach ( Elo and Kyngas, 2008) in the reading to identify passages that addressed an ambiguous circumstance or that reflected student uncertainty. Each of these passages was closely read, and codes created to signify the students’ own words. For the first 90 essays, the researchers worked independently in coding and then discussed each code until consensus was reached. Thereafter, each author took a different set of essays and passages and coded them independently with discussion following of new codes found, subtle additions to existing codes, or especially dramatic verbatim quotes that illustrated a code. This component of the analysis followed a traditional qualitative thematic analysis with constant comparison checking.

We each then independently arranged our codes into themes and, because of the structured content approach, used as the organizing theory that of transformative learning. Our themes (axial codes) became dilemma, transformative learning, and strategies. One further theme emerged as we studied our codes and Mezirow’s definition, that of the integration of the new learning/strategy into the ongoing life/practice of the student.

## Results/Analysis

Using the structured content approach, we were able to describe 4 themes within the students’ essays that discuss uncertainty and ambiguity. Applying the 10 items described by Mezirow:

A. The circumstance under which the student encounters ambiguity and uncertainty is Mezirow’s “ *disorienting dilemma*”.

B. The students’ *reactions* are the “self-reflection, critical assessment of assumptions, exploration of options for new roles, relationships and action.”

C. The students’ “toolbox” of *strategies* to cope with the ambiguous situation and their own uncertainty is “planning a course of action, building competence”

D. There follows *reintegration* based on new perspective.

### A. DISORIENTING DILEMMA

The disorienting dilemmas occurred when the clerk encountered clinical ambiguity in patients’ diagnosis, treatment, and/or prognosis. The disorienting dilemma, in part may have resulted from previously held assumptions. Clerkship year may be the first time they are confronted with the “vastness of the gap in where [they] currently [are] and where [they] want to be.” Through working with patients in a clinical setting, students find that “life as a student professional is no longer simply an academic exercise.” In transitioning from a purely academic classroom to the wards, they are confronted with certain truths: “medicine isn’t black and white,” “life isn’t fair,” among others. Patients do not present like neat and tidy multiple-choice questions, and they learn the full meaning of “patient’s do not read the textbook.” These common sayings all point to a broader discomfort and internal tension that results from clinical uncertainty. On a simpler basis, some students discuss awkwardness with not knowing how to communicate with patients and discover that patients often expect answers.

#### Clerkship is the disorienting dilemma

“When I began medical school, pre-clerkship (in my opinion) was straight forward..The task was clear, study the material the school provides, I’m then tested on said material and the world is easy. However, with clerkship changing that dynamic, there is no set material from which to master.I struggled initially, particularly in my first round, on coming up with a study plan that was realistic”

#### Medicine is an art

“There is no textbook way to do things, medicine it artful and scientific and while I am excited I am also terrified to hone my craft.”

“It wasn’t until clerkship year, .. I realized that medicine is one big educated guess.”

#### Patients don’t read the textbook

“Patients rarely present like a NBME style question,and I struggled with connecting the dots between patient stories, physical exam findings, differential diagnoses, and insurance coding.”

“My hands would start sweating and I would start thinking of the different differentials based on the nurse’s initial interview that I could report to my preceptor. This nervousness stemmed from my lack of patient interaction and the difficult encounters I had with some of the simulation patients during pre-clerkships. ..Going into clerkships, I thought real patients also would be like the simulated patientsand not open up unless I asked questions in a specific way.”

### B. STUDENT REACTIONS: TRANSFORMATIVE LEARNING

In our data set, we found that students reacted to the disorienting dilemma through transformative learning. The transformative learning occurred when the student discovered and dealt with the ambiguity and uncertainty through various metacognitive processes. Uncertainty forced some students to deeply reflect on their self-identity and how they approach problem solving. While some students adopted a more algorithmic style of thinking towards patients and disease processes, others found that algorithmic processes did not always apply to a given situation and could not replace clinical reasoning. Many students discussed the utility of the differential, a concept that they had been taught in pre-clerkship setting but did not completely understand how to use until clerkship. Students additionally developed a larger understanding of the concept of team of healthcare providers and learned that “ *patient care is not solely carried out by the physician,”* but rather includes nurses, medics, techs, social workers, and other healthcare professionals. As one student writes, *“it is impossible to be all knowing when it comes to medicine, and much of being a physician is working as a team and figuring it out.”* Although many students reacted to uncertainty through changing the way they thought, some found that uncertainty led to worsened self-image and began to question their own intellectual adequacy. However, for some students,, uncertainty shaped the way they saw themselves and found that it was okay to admit that they did not always know the answer. In dealing with clinical uncertainty, some students spoke about a change in perspective. In regards to dealing with a case of nonaccidental trauma, one student writes *“Learning to deal with situations like this has taught me a great deal about being a good provider as well as broadened my approach to life. I learned that although we want to interject our strong emotions into the situation of abuse, that is not our role.”*

#### Introspection

“Since beginning clerkships, I have found myself examining my thoughts, actions, and words more often than I have in the past.”

“Coming out of pre-clerkship, creating differential diagnoses was a weakness of mine. I was not motivated to practice this skill because I thought to myself, if I get the question right and know the diagnosis, why does the other stuff matter? It’s still a working progress, but I am more motivated now because I understand that it’s not about getting the answer right. The goal is to create comprehensive care for our patient which can only be done if we include the possibility that we are wrong and that we are prepared to swiftly correct our mistakes.”

#### Change in self identity

“I continue to ask myself, in these last 12 months: is it WORTH it? Should I consider another path? Is it reasonable to ask this question to myself? Are others asking the same question? Is the nobility of profession, and the ability to help others in this art of medicine worth sacrificing the many years of my life I have begun to shave off at the back end with my stress, weight gain, poor sleep and lack of family interaction?”

#### Algorithm

“There are evidence-based ways of handling many of the patient’s symptoms but there is no algorithm to treating the patient as a whole or making the most of the time she and her parents have left together.”

### C. STRATEGIES

The “toolbox” of strategies was developed by students as ways to cope with and hopefully combat uncertainty. The strategies included trusting the process of medical education, accepting responsibility of lifelong learning, learning how to use outside resources, and how to better communicate with patients to cope with uncertainty. They acquired skills needed to form the differential, such as asking the right questions, completing a thorough history and physical, understanding pathophysiology, and learning how to properly collect and synthesize information. The differential diagnosis was seen as an *“educated guess*” rather than an absolute truth and was a document to be reworked as new information became available. They learned to use outside resources such as guidelines, outside reading material, and how to better utilize the team of healthcare providers. Communication strategies developed, and some students learned how to admit “I don’t know” to patients as well as to staff. Some students started to shift to a patient centered outlook.

#### Communication

“I became much more comfortable with the response ‘I am not sure but I will look it up and get back to you.’”

#### Modeling senior staff

“I realized that even the attending physicians constantly are learning..”

#### Trust the process

“Since starting clerkship and preparing for the shelf exams I’ve learned the universe of medical knowledge far exceeds anything that I could hope to cram into my head to prepare for some exam. I am better at accepting that and realizing that it doesn’t mean I’m behind or I’m not good enough to be a physician. It means that becoming a physician is a process-a very long one, likely unequaled in any other profession. But there is a process and I’m on track and I need to trust the process.”

#### Use outside resources such as guidelines

“Lately l feel that a concept I’ve been able to understand better is the function of guidelines and the importance of the education we get as physicians. For instance, yesterday I reviewed GBS+ status in mother of newborns yesterday and how we have guidelines that are helpful but do not cover every situation that we will encounter and how basic science allows us to make clinical decisions that would otherwise be very difficult to make..”

### D. INTEGRATION

Students used the strategies they had developed, integrating them into their daily practice of medicine and into their reasoning patterns. They became “consciously competent” in dealing with clinically ambiguous situations. By doing so, they found they could tolerate the uncertainty and regained self-confidence. Behavior changes occurred as a result of implementing the strategies to cope with uncertainty.

#### Changes in attitude

“I realized that being incorrect or not knowing an answer was not a particularly bad thing; simply meant I was going to learn more that day. .. I stopped feeling guilty about not knowing everything and realized it was impossible”.

#### Being prepared

“Sometimes, the physician must make life or death decisions in a short amount of time. All these aspects of being a physician place large amounts of responsibility over our shoulders. We must prepare enough so that when the time comes we can shoulder that load, for the patients/nurses/techs look to us for the answers. Understanding and upholding that responsibility by being prepared is an important concept to being a physician.”

#### Working in a team

“I have learned a lot about medicine, but more importantly I have learned that I will never know everything. This is both a motivator to keep learning from every patient encounter and a great relief since it is okay not to be the expert on every topic. That’s because medicine is a team sport. I have found that knowing the right resources and people can be more effective than memorization. This is also a great approach to life as networking can often get you farther than individual effort alone.”

“it is impossible to be all knowing when it comes to medicine, and much of being a physician is working as a team and figuring it out.”

#### Patient-centered approach

“My thought processes have shifted from a self-centered approach, for lack of a better word, to a patient centered approach that uses me and my knowledge as a voice for that patient’s education.”

#### Use of time

“being well prepared for the medicine I was going to see in clinic the next day was more important than adding those 10 extra practice questions.”

#### Studying

My “new approach” to learning is less of a new strategy and more of a new attitude. It is not just that I read every day now, but that I read with a hunger for knowledge. For example, when I read about asthma, I used to ask myself, “On a test, what do they ask about asthma and what are the right answers?” Now I ask, “How would I distinguish an asthma exacerbation from pneumonia or foreign body aspiration?”

## Discussion

Mezirow’s Transformative Learning Theory proved a useful lens to examine students’ encounters with uncertainty in clinical practice. According to Mezirow,

“transformative learning is the process of effecting change in a frame of reference..Self-reflection can lead to significant personal transformations..Transformations in frames of reference take place through critical reflection and transformation of a habit of mind..We do not make transformative changes in the way we learn as long as what we learn fits comfortably in our existing frames of reference.” ( Mezirow, 1997).

In the case of the data shown here, students were able to show changes in perspective and evidence of transformative thinking as they grappled with their disorienting dilemmas. Some students found that their frame of references were challenged and incorporated changes in their self-identity, thus demonstrating the beginning of professional identity formation. In adopting new communication strategies, students were able to say “I don’t know” because they realized they are still learning and value honesty in the patient-provider relationship. In doing so, they were beginning to shift from provider-centric to patient-centered care. Our results are consistent with previous studies on medical students’ encounters with uncertainty and further enrich our understanding. ( Fox, 2000; Merrill, 2014).

The disorienting dilemma can be a challenge to the students’ identity as a ‘smart’ person which often leads to transformative thinking, a critical self-appraisal and adoption of new metacognitive processes such as accepting the discomfort of not knowing the correct answer. As they begin to assess their perspective, they begin to develop a toolbox of strategies, and may choose how to integrate these strategies into their clinical practice and life. However, this progression is not a simple unidirectional process. Concepts such as the team were both evidence of transformative thinking and a strategy to cope with uncertainty. Once students have developed such strategies, they may begin to employ and integrate these new strategies into their life and incorporate them into their practice.

In each set of essays in the data, there was evidence of the disorienting dilemma, transformative thinking, and development of a toolbox of strategies. As could be expected from a diverse medical student class that has a wide breadth of ages and experiences, some essays showed a deeper depth of knowledge and perspective than others. This is interpreted by the researchers as that the students will progress through the transformative thinking at different rates and adopt strategies of coping with uncertainty at different times. To borrow a common phrase from the students themselves, students also don’t read the “textbook” in terms of learning and metacognitive thinking.

### Strengths and limitations

Results are limited to the population of one medical school, and this may not be generalizable given the uniqueness of our military medical school. The data set was robust in that it covered two entire medical school classes throughout two separate clerkship years.

### Next Steps

In this study, the temporal aspect of progression through steps of transformative learning was not studied given that aspects of the disorienting dilemma, transformative thinking, and adoption of strategies were seen in all data sets. Future studies of the data may reveal a progression of adoption of strategies and depth of critical thinking. In future iterations of the reflective practice essays, the question probes will be changed to become more targeted regarding the concept of uncertainty and ambiguity to better assess how students are dealing with these topics. Adoption of focus groups could also add another enriching data set to show how students are coping with uncertainty. Focus groups may additionally add a therapeutic as well as diagnostic approach as in our data set it was found that realizing others were dealing with uncertainty helped students. At this medical school, students discuss their uncertainty papers during preceptor led sessions at the clerkship site, however this information is not currently recorded or used.

## Conclusion

We found Mezirow’s theory of transformative learning to be a useful framework for a structured content analysis of medical students’ learning about uncertainty. Learning begins with a disorienting dilemma, for our students it was often a feeling that they should know something that they don’t know. Transformative learning took place as the student self-reflected on the dilemma and began to see it in a wider scope. Students developed strategies with which to become comfortable with the dilemma (often adopted from watching residents and faculty deal confidently with the dilemma that has shaken the students’ self-confidence). Integration takes place when the student uses the newly acquired strategies in their clinical practice.

## Take Home Messages

• Students learn how to deal with uncertainty by observing residents and attendings and adopting approaches to clinically ambiguous situations that have been modeled.
• Students learn that it is okay to say “I don’t know” and become comfortable with their own uncertainty.
• Students actively acquire skills to formulate a differential diagnosis that addresses uncertainty. They begin to understand that the differential is a working document that includes the possibility that it is wrong.

## Notes On Contributors

Charisse Villareal is a 4 ^th^ year medical student with an undergraduate degree in chemistry. Now, at the beginning of her medical career, she approaches ambiguous situations by reading more, attempting to increase her knowledge base, and seeking help when she feels out of her depth.

Virginia Randall is a senior pediatrician with 30 years of experience in treating infants and toddlers with disabilities. She approached ambiguous situations, her own and parents’ uncertainty regarding diagnosis and prognosis, by ensuring frequent follow-up visits with the patient, often including home visits. This made the uncertainty easier to bear, as joint observations and thoughts were transparent. ORCID iD: https://orcid.org/0000-0003-2944-9015

## Declarations

The author has declared that there are no conflicts of interest.

## Ethics Statement

The study was determined as Exempt Protocol PED-86-4343, eIRB ref #913361. The Amendment 12 for your EXEMPT human subjects’ research protocol PED-86-4343 entitled, “Exploring Threshold Concepts in Medicine through Medical Student Reflective Practice Essays," was reviewed and determined on April 16, 2019 by Sharon M. Randall, Exempt Determination Official to not affect the original exemption of this protocol under the provision of 32 CFR 219.101(b) (l). Institutional Review Board, Uniformed Services University of the Health Sciences.

## External Funding

This article has not had any External Funding
