# Supplementary material for: "It's Okay to Say 'I Don't Know'": Medical Students Use Transformative Thinking to Cope with Ambiguity and Uncertainty
Source: MedEdPublish (2016). 2020 Jan 15;9:14. [Version 1] doi: 10.15694/mep.2020.000014.1 (PMC10702687; doi:10.15694/mep.2020.000014.1)
Supplement: Peer review history

# Reviewer report: Reviewer response for version 1

**DOI:** 10.21956/mep.19973.r30365

**Published:** 2020-06-28

**Authors:** Megan Anakin

**Affiliations:** 1 University of Otago

This review has been migrated. The reviewer awarded 3 stars out of 5 Thank you for publishing this article. I am working on this topic with colleagues and I read your article with great interest. The introduction provides an overview and definition of uncertainty including a model for understanding phases of learning about uncertainty. The second to last paragraph in the introduction about Threshold Concepts is tangential to the focus of the article since the authors do not pursue this theoretical framework in their study. The authors may wish to omit this paragraph to make room for other background information. Missing from the introduction is background information about other studies that provide the rationale for the current study. The authors may wish to state the aim of their study at the end of the introduction to help the reader better appreciate the alignment of the methods chosen and their use of Mezirow’s ideas as the theoretical framework for this study. In the last two sentences of the methods, the authors begin reporting their results. Please consider moving the reporting of themes to the results section. Please also considering moving analysis procedures in the results section into the methods and renaming the third section ‘results’ only. Please consider explaining how the 10 phases of learning were reduced to four items listed in the first paragraph of the results in the methods section. When reporting quotations from participants’ essays, please provide an attribution. Please explain the process of randomly assigning an anonymous identification code to each essay to protect the identity of the participants in the methods section then use the codes when reporting quotations in the results section. This detail will address the readers’ concern that all quotations are unattributed so that there is no way of knowing if all quotations are from one source, a limited number of sources, or from a wide range of essays. This important step will enhance the trustworthiness, robustness, and representativeness of your findings. Please consider explaining the headings placed before the quotations. The reader needs more information to make sense of the quotations that are provided outside of the main text. Please note that the subsection heading ‘D. Integration’ does not match the name of this theme stated in the first paragraph of the results (i.e. ‘reintegration). This mismatch should be corrected. The authors may wish to enhance the first two paragraphs of their discussion section by reminding the reader how the findings address the aim of the study and by emphasising the features of the findings that further our understanding of how students cope with ambiguity and uncertainty. Please consider enhancing the strengths and limitations section of the discussion by following the advice from Lingard, L. (2015). The art of limitations. Perspectives on Medical Education, 4(3), 136-137. I look forward to reading the second version of this article.

Reviewer Expertise:

NA

No decision status is available

# Reviewer report: Reviewer response for version 1

**DOI:** 10.21956/mep.19973.r30364

**Published:** 2020-01-24

**Authors:** P Ravi Shankar

**Affiliations:** 1 American International Medical University

This review has been migrated. The reviewer awarded 5 stars out of 5 I enjoyed reading this research paper. The authors have described third year medical students’ reaction to and coping with clinical uncertainty and ambiguity. The transition to the clinics makes learning and knowledge more uncertain. The basic sciences are predominantly university and class room based learning and is more structured. Clinical learning depends on a number of circumstances and can be less structured and organized. I distinctly remember this during my transition from the first phase of the undergraduate medical program to the clinical phase. The authors have described the methodology well. The limitations have also been mentioned. The process of transformative learning has been addressed. I am happy to note that a medical student is a co-author of the paper. As the authors mention some aspects at the Uniformed Services University may be different from those at other medical schools.

Reviewer Expertise:

NA

No decision status is available

# Reviewer report: Reviewer response for version 1

**DOI:** 10.21956/mep.19973.r30368

**Published:** 2020-01-18

**Authors:** sathyanarayanan varadarajan

**Affiliations:** 1 SRM Medical College, SRM University

This review has been migrated. The reviewer awarded 5 stars out of 5 This well-written article is based on a Qualitative study done by analyzing a robust 273 reflective practice essays from pediatric clerks from academic years 2016 and 2018 for encounters with clinical uncertainty and ambiguity and examined how they reacted to these dilemmas.This article explores new dimensions in the students’ perceptions on uncertainty and ambiguity and adoptive mechanisms in tackling these challenging situations. By using the structured content approach, they describe 4 themes within the students’ essays that discuss uncertainty and ambiguity. They also used Mezirow’s Transformative Learning Theory to examine students’ encounters with uncertainty in clinical practice.They noticed that students reacted to the disorienting dilemma through transformative learning. The transformative learning occurred when the student discovered and dealt with the ambiguity and uncertainty through various meta-cognitive processes.This article will be helpful for all the medical educators who are involved in training medical students and particularly Interns. It’ll be of immense value in helping them to understand clinical situations of uncertainty and dilemma and solve them with more self-confidence. The authors also highlighted on the Strengths and limitations as well as next steps.I agree with the authors totally that “It's Okay to Say 'I Don't Know'" …as it can lead to transformative learning…!!

Reviewer Expertise:

NA

No decision status is available

# Reviewer report: Reviewer response for version 1

**DOI:** 10.21956/mep.19973.r30366

**Published:** 2020-01-15

**Authors:** Felix Silwimba

**Affiliations:** 1 University of Lusaka

This review has been migrated. The reviewer awarded 5 stars out of 5 I like this purely qualitative study on medical student interaction and experiences with real patients. the study is realistic and relates to the truths about clerkship. I recommend this study

Reviewer Expertise:

NA

No decision status is available

# Reviewer report: Reviewer response for version 1

**DOI:** 10.21956/mep.19973.r30367

**Published:** 2020-01-15

**Authors:** Janie Dade Smith

**Affiliations:** 1 Bond University

This review has been migrated. The reviewer awarded 5 stars out of 5 This is a really interesting study with really interesting results. It made me think we could do this at our university.

Reviewer Expertise:

NA

No decision status is available
